# The impact of Sino-foreign cooperative universities in China on Chinese postgraduate students’ learning performances in the United Kingdom and United States

**DOI:** 10.3389/fpsyg.2022.1012614

**Published:** 2022-10-11

**Authors:** Bin Zou, Xueying Wang, Chunmin Yu

**Affiliations:** ^1^Department of Applied Linguistics, Xi’an Jiaotong-Liverpool University, Suzhou, China; ^2^Suzhou Foreign Language School, Suzhou, China

**Keywords:** EMI, Sino-foreign cooperative universities, learning experience, non-EMI, China

## Abstract

Sino-foreign cooperative universities in China provide English-Medium instruction (EMI) and enable students to have an international education mode. In contrast, Double First-Class universities provide non-EMI education mode in China. After graduation from their undergraduate studies in China, many students go to the United States or the United Kingdom for their postgraduate studies. However, very few studies have discussed the impact of the EMI context in Sino-foreign cooperative universities and compared students’ learning experiences in the EMI context with the non-EMI context in China. Therefore, this study aimed to explore students’ perceptions of their learning experience from the two contexts and their impact on their postgraduate study in British and American universities. A combination of quantitative and qualitative data collection including questionnaires and interviews was adopted in this study. The findings show that students’ learning experience in the EMI context seems to benefit more than students who are from non-EMI context when they strived for their postgraduate studies in British and American universities.

## Introduction

China’s government has encouraged international collaborations between Chinese universities and universities in western countries to set up Sino-foreign cooperative universities and degree programmes in China since 2004. Nine Sino-foreign cooperative universities have been established and delivered degree programmes in the English-Medium instruction (EMI) mode, such as Xi’an Jiaotong-Liverpool University (XJTLU), Nottingham Ningbo University, Shanghai New York University, etc. These Sino-foreign cooperative universities provide students with a different learning experience including EMI context, adhering to curriculum design, teaching and assessment standardizations from cooperative foreign universities, and more foreign staff members than Chinese staff members, compared with other traditional universities in China (Zhao, 2021). Many Chinese students go to Anglophone settings for their postgraduate studies after graduating from traditional universities or Sino-foreign cooperative universities. According to the China National Bureau of Statistics (National Data, 2021), 662,100 Chinese students sought out education abroad in 2018, three times higher than in 2009 (229,300). In addition, Chinese students have become the largest and fastest increasing group of international students in the world (Cebolla-Boado et al., 2018) since they are attracted by various factors, including language learning contexts, quality education, career prospects, and authentic foreign cultural environment (Chen and Zimitat, 2006).

Therefore, more studies should be conducted to examine the impact of the EMI context in China on Chinese students’ learning experience in their postgraduate studies abroad. Furthermore, comparison studies between Sino-foreign cooperative universities (EMI context) and non-Sino-foreign cooperative universities (traditional and non-EMI context) in learners’ learning experience abroad could be investigated further. Because many Chinese students decide to go overseas for graduate studies compared to undergraduate students, this research intended to explore Chinese postgraduate students’ learning experience in Anglophone university settings, particularly in the United Kingdom and the United States. Specifically, students who were graduated from Double First-Class universities (top universities in China and non-EMI context) and XJTLU (a Sino-foreign cooperative university and EMI context) in China were selected to compare learning performances between traditional universities in China and Sino-foreign cooperative universities. It attempted to look at the potential influence of reforms in international collaborations in running Sino-foreign cooperative universities in China.

## Literature review

### Sino-foreign cooperative universities

Internationalization is indispensable in China’s modernization of higher education (Li, 2015). As a new form of higher education, transnational higher education has become a common trend in China. It cooperates with foreign and Chinese educational institutions to set up projects or institutions in China, mainly providing educational services to Chinese citizens (The State Council of the People’s Republic of China, 2020). In addition, Sino-foreign cooperative universities are a significant feature of higher education internationalization and the modern university system (Wang et al., 2020). Until June 2021, the total number of Sino-foreign cooperative universities approved to be established or held nationwide is nine (Wang, 2021).

XJTLU, as a Sino-British university, was formed in 2006 from the partnership between the University of Liverpool, United Kingdom and Xi’an Jiaotong University, China. It aims to educate “technical and managerial professionals with international perspectives and competitive capabilities” (XJTLU, 2017), and both parent institutions shared control of their curriculum. The goal of XJTLU is to adapt to the trend of higher education reform, integrate the world’s excellent educational practices and resources, and cultivate talents with global competitiveness according to future social trends and requirements (Hui, 2021). In XJLTU, English is the medium of instruction in which students receive English learning and teaching context for all subjects. In addition, most students select to study further overseas after graduation. According to XJTLU’s annual report on careers and employment in 2021, 85% of students went overseas to look for a master’s degree. 33% entered the Top 10 universities and 85% entered the Top 100 universities for postgraduate studies (XJTLU, 2022).

### Double first-class universities in China

Generally, First-class universities and disciplines of the world are referred to as “Double First-Class universities” in China. It is national strategy in China’s higher education field after the “985 Project and 211 Project,” which is conducive to enhancing the comprehensive strength and international competitiveness of China’s higher education (The State Council of the People’s Republic of China, 2015). Concretely, the list of Double First-Class universities was officially announced in 2017, with 137 construction universities and 465 construction disciplines, representing top universities and top subjects in China (Ministry of Education of the People’s Republic of China, 2017).

Furthermore, Chinese students should undertake the National College Entrance Examination (Gaokao) to apply to universities according to their scores. In 2011, for instance, the total College Entrance Examination mark in Jiangsu Province was 480, and the Tier One (the first level) university for science students was 345; the Tier Two (lower than Tier One) was 320. The lowest entrance line of most Double First-Class universities for Jiangsu students often far exceeds the Tier One universities. Taking Nanjing University as an example, in 2011, its lowest entrance line for science students was 382, 37 points more than the Tier One. As for XJTLU, in 2011, the lowest entrance line for science students in Jiangsu Province was 345, which met the first broad line (Jiangsu Entrance Examination, 2011). Therefore, most students who entered Double First-Class universities in 2011 will likely have higher scores than XJTLU.

XJTLU and Double First-Class universities are different in terms of several aspects, such as EMI context (XJTLU), non-EMI (Double First-Class universities) context, education philosophy, curriculum design, learning contents, and pedagogical methods. Another main difference is students’ English courses in their first and second years. As Huang (2017) mentioned, in most ordinary Chinese universities with non-EMI, the College English course is General English and students take General English courses for 1 or 2 years. However, general English skills may not be significantly helpful to EFL learners learning achievements in the EMI context (Curle et al., 2020). Whereas, XJLTU students must take an English for Academic Purposes (EAP) course to cope with their studies in the EMI environment. It is demonstrated that students learn the vocabulary and academic skills they need to know in their disciplines, such as paraphrasing, reference and citation, critical thinking, and academic conventions (Li and Ruan, 2015; Zou and Jiang, 2021). Soruç et al. (2022) addressed that academic English proficiency can help students have better learning performance in the EMI context. Furthermore, Wu and Zhang (2017) found that EMI environment might help EFL students achieve better writing competence than EFL students in a non-EMI environment.

### Chinese postgraduate students studying abroad

Overall, research about Chinese students studying abroad is not rare. Since there is an increasing number of students who choose to pursue higher education in foreign countries, Counsell (2011) conducted a self-report questionnaire survey among 188 Chinese students who are final-year undergraduates or postgraduates in two British universities. It is found that their pursuit of better higher education quality and their desire to improve English language competency and skills motivated them to study abroad. In addition, they considered British universities’ degrees as containing higher value than Chinese degrees (Counsell, 2011). However, barriers exist on their way to pursuing the degree, especially in language. Specifically, according to Hu (2014), the language problems of Chinese international postgraduates mainly focus on the lack of verbal expression ability, listening training, and the cultivation of academic writing ability. Moreover, Huang (2017) investigated the English learning strategies of 60 Chinese students for master’s degrees in American universities through questionnaires and interviews, proving that foreign language learning environments and different English proficiency can lead to different utilization of learning strategies.

In addition to the language barrier, critical thinking ability is also a learning obstacle for Chinese international students. A comparative study of critical thinking between domestic and overseas postgraduates confirms that postgraduates who have studied in the United Kingdom after 1 year of postgraduate study present better critical thinking ability than domestic postgraduates (Cheng, 2017). Additionally, Wu (2015) conducted a case study of the learning conditions of mainland Chinese students who are studying in British universities, and concluded that how Chinese postgraduates approach their learning are closely related to many factors, containing culture, learning beliefs and behaviors, academic contexts, and intellectual development.

Furthermore, some studies focused on the intercultural adaptability of students abroad. Concretely, Xiao (2018) explored the differences and challenges faced by Chinese students in their study and life in the United States with the help of cross-cultural adaptation theory and put forward some practical suggestions on the cultivation of the cross-cultural adaptation ability of Chinese abroad students. Yan (2011) also discussed the cross-cultural adaptation and challenges of Chinese students in the US from a cross-cultural perspective. Through the qualitative data of interviews, he gives feedback that the challenges faced by overseas students mainly come from their learning tasks, cultural adaptation, and personal development. Lu and Li (2012) adopted the cultural sensitivity scale to quantitatively evaluate the intercultural sensitivity of 100 Chinese postgraduates at home and abroad. They conclude that the cultural communicative competence of Chinese postgraduates studying abroad is higher than domestic postgraduates.

### Previous studies on learning outcomes from studying abroad

The evaluation of learning outcomes is often used in international higher education, and the literature related to learning outcomes is usually discussed with various features, such as learning satisfaction, learning skills, and learning experiences (Purdie and Hattie, 1999; Johnson et al., 2000). When assessing the general pedagogical outcomes, three broad criteria need to be involved: critical literacy skills, including communication, critical thinking, problem solving, and interpersonal skills, and citizenship skills, including community involvement, intercultural understanding, and leadership (Alfred et al., 1999). As a result, some of the features above are also of high importance when studying overseas postgraduates’ learning outcomes. For instance, critical thinking is a vital component; as Major (2005) mentioned, western teaching and learning culture concentrates on the drilling of criticizing, questioning, and debating. In addition, transferring from the Chinese learning environment to the United Kingdom also concerns students’ community involvement skills and intercultural understanding abilities (Major, 2005).

As a unique group, Chinese international students’ ability to solve problems in a new foreign environment and adaptability to social interaction has become a significant trend in studying abroad (Comp et al., 2007). Concretely, the learning achievements of international students are sufficient, since Sutton and Rubin (2004) claimed that the participants who study abroad are more proficient in global cultural knowledge, international professional knowledge acquired through foreign academic learning (Meyer-Lee and Evans, 2007), and the improvement of corresponding language skills (Murphy-Lejeune, 2002). Li (2017) conducted a qualitative study by interviewing Chinese learners studying in Germany and found that improving learning outcomes benefits from group cooperation and discussion in a foreign context. Through face-to-face interviews with 15 Chinese postgraduate students studying in the United Kingdom, the results show that English proficiency level is an essential factor affecting the learning outcomes of international students (Zhang, 2017). Similarly, Soruç et al. (2022) also found that academic English proficiency significantly benefits students’ learning achievement in an EMI environment. Besides, it is demonstrated that through self-assessment of the global learning experience, international and transnational learning outcomes will contribute to expanding the employment prospects of Chinese students and help their career development (Mok et al., 2018).

However, although studies can be found on students learning outcomes in Sino-foreign cooperative universities, or Double First-Class universities, and Chinese postgraduate students learning outcomes in the Anglophone settings, research comparing similarities and differences in learning outcomes between graduates from Sino-foreign cooperative universities and graduates from Double First-Class universities in their postgraduate studies still needs further investigation so that the impact of internationalized innovation in higher education in the Sino-foreign cooperative universities in China can be explored with more evidence. Therefore, it is interesting to investigate the impact of Chinese-foreign cooperative universities on China’s internationalization of higher education compared to traditional universities in China. Since there are only a few Sino-foreign cooperative universities in China, this study selected XJTLU—an EMI context, as representative Chinese-foreign cooperative universities, and Double First-Class universities to look at whether there are differences in their graduates’ learning performances in postgraduate studies in some Anglophone universities. This research focused on universities in the United Kingdom and the United States because the United Kingdom and the United States are the top two countries for XJTLU’s graduates to study overseas for their postgraduate degrees (XJTLU, 2022). Therefore, this research is aimed at addressing the following questions:

1. What skills should students acquire to help their postgraduate study in universities in the United Kingdom and the United States?
2. What are the similarities and differences in learning styles between XJTLU graduates and Double First-Class university graduates in postgraduate study in universities in the United Kingdom and the United States?

## Methodology

### Participants

One-hundred seventy participants who were postgraduate students in the US and the United Kingdom were randomly selected to participate in this study, and they all acquired bachelor’s degrees from XJTLU or Double First-Class universities. Specifically, among all questionnaires, there were 90 valid responses from British postgraduates and 80 valid responses from American postgraduates. Furthermore, 79 respondents graduated from XJTLU and 91 from Double First-Class universities. XJTLU was coded as XPS and Chinese Double First-Class universities were coded as CPS. 65% of the participants are female and 35% are male. The snowball sampling strategy was also used to take advantage of the existing social networks among the target groups (Cohen et al., 2007). Thirty volunteer postgraduates from those who had completed the online questionnaire were invited to participate in the semi-structured interview. Thirty were from the EMI context and 17 were from the non-EMI context.

### Instrument

This research adopted mixed methods to collect and analyze data since the participants’ authentic perspectives can be presented through quantitative and qualitative research methods (Cohen et al., 2007). Firstly, the questionnaire was designed based on literature and facts about Chinese students abroad including learning satisfaction, learning skills, and learning experiences (Purdie and Hattie, 1999; Johnson et al., 2000), language competence (Hu, 2014), learning environments, learning strategies and different English proficiency (Huang, 2017; Zhang, 2017; Soruç et al., 2022) and critical thinking (Major, 2005; Cheng, 2017). It was distributed by an online questionnaire survey platform *Wenjuanxing*. Subsequently, in order to address more concrete research questions, follow-up semi-structured interviews were carried out based on the survey results.

The questionnaire consisted of four main parts. The first part is the basic demographic information, including gender, grade, discipline, undergraduate school and postgraduate university. The second section focused on self-evaluation of postgraduate students’ learning satisfaction. Students were asked to evaluate their learning satisfaction in their postgraduate studies in the United Kingdom and the United States. The third part concentrated on comparing undergraduate and postgraduate teaching styles, pedagogies, curriculum design and assessments, etc. The final area focuses on learners’ mastery of relevant study skills including critical thinking, research skills, language competence, etc. In addition, the questionnaire had been tried and modified to achieve validity and reliability before wide distribution. The Cronbach’s alpha of each factor was more than 0.5 but less than 0.6. However, as Qin (2009) argued that 0.5 could also be acknowledged when the number of items is not large. Since the number of the questionnaire in this study was only 170, the items with Cronbach’s alpha over.5 were all retained. Meanwhile, the KMO for all items was 0.7, which indicated that these items were appropriate for factor analysis and had good reliability and validity (Pallant, 2020).

The questions of this semi-structured interview are mainly divided into three parts. To be more specific, the first part is learning performance, including the outcomes and reasons for postgraduates’ performance, useful learning skills, and adaptability to different learning environments. The second section is about learning satisfaction, exploring students’ problems in learning abroad. The third area focuses on participants’ learning behavior and style, seeking to understand the similarities and changes in learning styles in the undergraduate and postgraduate periods of XPS and CPS.

### Data analysis

In the questionnaire, tables and forms were generated to compare CPS and XPS and to explore the correlations between factors raised in the questionnaire, such as learning satisfaction and motivation to go abroad, English language competence, and undergraduate learning outcomes. Additionally, some extracts from follow-up interviews were provided in the quantitative analysis to explain some phenomena.

Concerning the interview’s analysis, each recording was transcribed into text after the interview. Each interviewee was given a pseudonym. Then a three-level coding system containing features from Grounded Theory (Dörnyei, 2007) to analyze the interview data after reading through them several times. In the initial open-coding level, codes and categories were assigned to the interview data segments to generate ‘meaningful chunks’ (Brice, 2005, p. 163); for example, labeling students’ some responses as ‘self-learning ability’. Subsequently, the second coding stage compared and contrasted the codes in the transcripts of each interviewee to find the relationship between them and then integrated them into more complex categories. Ultimately, the last selective coding section selected ‘core categories’ (Dörnyei, 2007, p. 261). For instance, ‘language competence’ was used as the central theme to include students’ speaking, listening, writing, and reading abilities. Furthermore, some supportive interviews extracted and paraphrased were attached for analysis under each theme.

## Results

### Comparisons of learning satisfactions and difficulties between CPS and XPS

**Figure 1:**
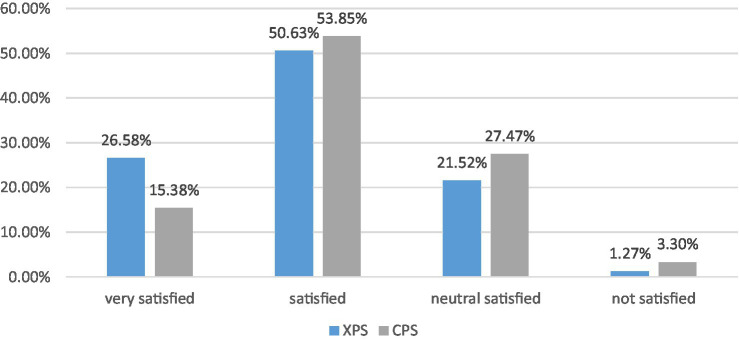
XPS and CPS postgraduates’ learning satisfaction.

Figure 1 below shows participants’ learning satisfaction with their postgraduate study in the United States and United Kingdom. Participants self-assessed their learning satisfaction, referring to affecting factors, such as scores they received in assignments, and class participation, on the scale by rating from ‘Not Satisfied at all’ to ‘Very Satisfied’. Overall, Figure 1 shows the result of XPS students’ satisfaction evaluation, and the degree of dissatisfaction of the two groups is deficient. Specifically, it can be seen that 77.21% of the 79 students are satisfied with their postgraduate studies, and 26.58% of them are very satisfied. Regarding the 91 CPS students (Figure 1), about 69% of them are generally satisfied with their postgraduate study, and only 15% are very satisfied. Although the Chi-square result shows that there is no significant difference (*p* = 0.209 > 0.05), the data comparison can still indicate that the overall learning satisfaction of XPS may be slightly higher than those of CPS.

**Figure 2:**
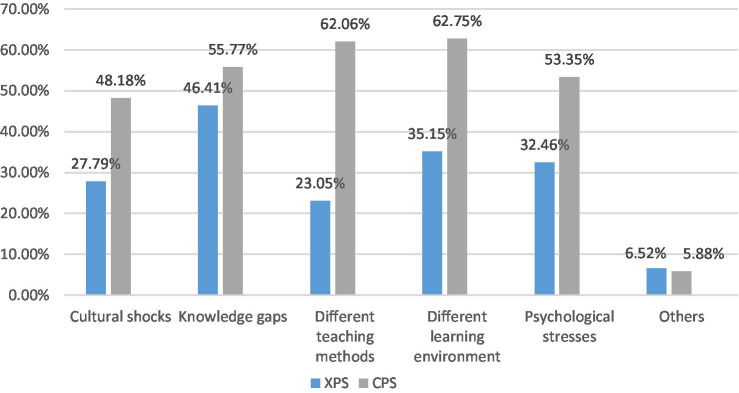
Learning difficulties CPS and XPS encountered during postgraduate study.

Figure 2 illustrates the different learning difficulties encountered by CPS and XPS during their postgraduate study, which could be the reasons for their different learning satisfaction, including cultural shock, knowledge gaps between undergraduates and postgraduates, different teaching methods, different learning environments, and psychological pressure. In general, participants can multiple-select the five hypothetical difficulties, and CPS has more difficult than XPS. More specifically, CPS encountered a significant challenge in the “different teaching methods compared with undergraduates,” which is about 39% more than XPS. Moreover, the Chi-square result shows that there is a significant difference (*p* = 0.022 < 0.05) in reported teaching methods in undergraduate studies between the two groups. Thus, it demonstrates great differences in teaching methods between Double First-Class universities and Western countries; therefore, postgraduates should adjust and adapt to the new teaching methods in a foreign context. Secondly, CPS also encountered some difficulties in cultural shock, different learning environments, and psychological pressure, while XPS has encountered fewer difficulties than CPS. In particular, the Chi-square result shows that there is a significant difference (*p* = 0.002 < 0.05) in reported different learning environments between the two groups. In addition, “knowledge gaps between postgraduates and undergraduates” exists the least gap, which illustrates that the knowledge and skills of postgraduates are more challenging than those of domestic undergraduates are.

Combined with the follow-up interviews, the reason for this difficulty is that there might be a considerable difference between the majors they study in undergraduate universities and the master’s programs they study in foreign countries. Tom, who majored in Economics and Law in Double First-Class university and graduated from Columbia University expressed dissatisfaction with his postgraduate learning: “My language background is weak; thus the language is the biggest challenge to me. And I changed my major, I feel the struggle. Finally, I am also confused about the curriculum arrangement.” Besides, a CPS student (Susan) in University College London (UCL) said, “I studied Chinese Language Teaching, and after graduating, I taught Chinese in a primary school in Nanjing for a year. Then you know now I’m working hard to get a master’s degree in History. However, this is not only about history. We even have classes about medicine, which really drive me crazy.” As a result, it is confirmed that these difficulties are the challenges for CPS postgraduate learning.

### Comparison between undergraduate and postgraduate studies

#### Differences between teachers

**Figure 3:**
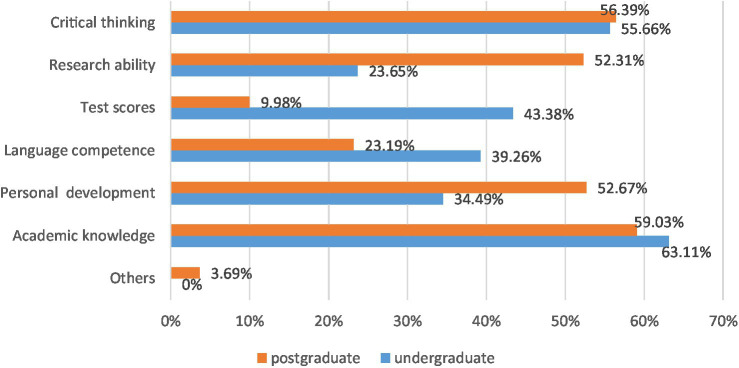
Comparison between factors emphasized by XPS teachers in Postgraduate study and undergraduate study.

In order to understand why there are significant differences in the challenges faced by the two groups, it should examine the key factors that instructors’ teaching focuses on during their undergraduate and master’s studies. When students were asked to select certain aspects which teachers mainly emphasized during their undergraduate and postgraduate study, a similar pattern could be found in XPS’s answers, as Figure 3 shows below. Two factors, which are “critical thinking ability” (postgraduate: 56.39%; undergraduate: 55.66%), and “academic knowledge” (postgraduate: 63.11%; undergraduate: 59.03%), are both considered as significant dimensions emphasized by teachers in their undergraduate and postgraduate study. It is indicated that when XPS finish their undergraduate study and start to take the postgraduate course, they might find it easier to get used to the postgraduate study and be well-prepared to achieve a better learning outcome. Hence, it could be one of the reasons why they were more satisfied with their current learning outcome. In addition, it can be seen from the graph that there are huge gaps between undergraduate and postgraduate teachers in terms of research ability, test scores, and personal development. Since undergraduate teachers are required to score the students’ tests and assignments, the whole score is used to apply for foreign postgraduate learning. Besides, during postgraduate study abroad, teachers often reduce examinations and pay attention to cultivating students’ research ability and developing personal ability.

Furthermore, there is a quotation from Jack from XPS who is now studying a master course at the University of Cambridge, United Kingdom: “Especially in the final year when I was doing final year project. Each week when I met my supervisor in the tutorial, he/she led me to develop critical thinking pattern and ability gradually.” Moreover, Mary, an XPS student from the University of Texas, United States, said: “critical thinking has been emphasized more during the undergraduate period. After all, it was a central idea that runs through English academic papers all the time; and the professor also emphasized this point. It should be more emphasis on postgraduate learning.” In summary, the content of the interview also confirms that critical thinking ability plays an irreplaceable role in postgraduate learning.

**Figure 4:**
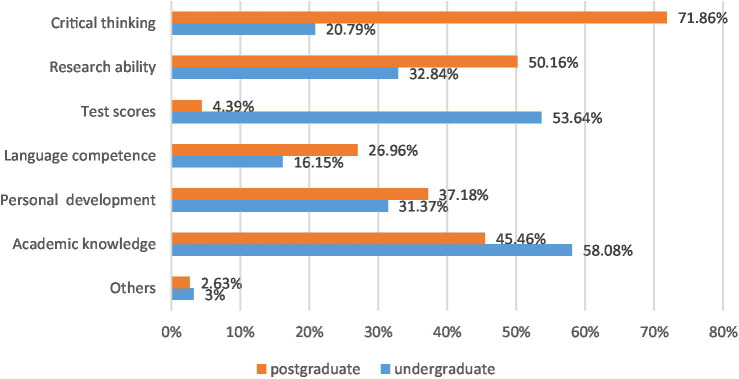
Comparison between factors emphasized by CPS’ teachers in postgraduate study and undergraduate study.

Whereas for students from CPS, it shows in Figure 4 below that there are various gaps between factors emphasized by teachers in their undergraduate and postgraduate courses. Specifically, CPS’s teachers in the undergraduate study focused more on “academic knowledge” (58.08%) and “test scores” (53.64%), and they cared less about “critical thinking” (20.79%). This factor is frequently mentioned by postgraduate tutors (71.86%). Additionally, the “critical thinking” data proved Major’s (2005) argument on the western culture of learning: classes are student-centered and more forms of discussion are available. Therefore, undergraduate and postgraduate teachers’ different focuses in pedagogy could impede students’ adaptation to their postgraduate courses in foreign universities, which reflects that a transition needs to be overcome. It is worth noting that comparing the data of XPS (Figure 3) and CPS (Figure 4), there is a large number gap in terms of critical thinking, which proves that the undergraduate teaching focus of XJTLU and Double First-Class universities have huge differences, and eventually generate two distinguishing effects on students’ postgraduate study abroad.

Furthermore, Mark from a CPS in UCL, United Kingdom said that the instructor sometimes considered that the content of the essay is superficial and general, without different personal opinions, which caused lots of pressure during postgraduate paper writing. Simultaneously, Jane, another CPS student at Columbia University, United States noted that the purpose of undergraduate learning is mainly for the final examination, and teachers rarely emphasize dialectical thinking. Thus, Chinese and Western pedagogical focuses have huge gaps, especially in cultivating critical thinking ability.

In summary, from the enormous differences between the two groups of figures, it is indicated that Double First-Class universities’ undergraduate study seems to pay more attention to the ability to pass the exam, rather than the cultivation of critical thinking; thus, such a learning model may generate students’ lose part of their innovative competence. On the contrary, XJTLU’s undergraduate education emphasizes pedagogical quality and the cultivation of students’ critical thinking, which is consistent with the educational philosophy of American and British universities. Especially its teaching system is full of international education characteristics, which expects students to cultivate better independent critical thinking and academic skills. Hence, XPS is easier to adapt to postgraduate study in foreign countries, and the proportion of difficulties and challenges is relatively insignificant.

#### Differences in curriculum designs

**Figure 5:**
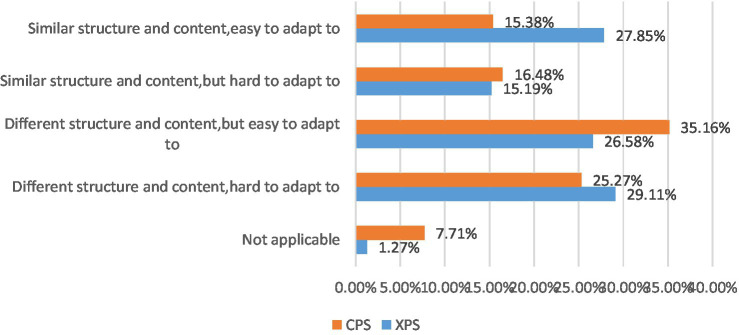
Comparisons between undergraduate and postgraduate curriculum designs of XPS and CPS.

In terms of comparisons between curriculum designs of the undergraduate universities and postgraduate universities, about 44% of XPS held the view that the structure and content are similar; even for the 26.58% of XPS who found the curriculum design was different, they felt that it was easy to get adapted to (Figure 5). Linda from UCL explained that “the postgraduate courses are very similar to those in XJTLU. Although the contents of the courses are different, the ways of teaching and grading are very similar, probably because they belong to the British education system. It does not take time to get used to them.”

Regarding those CPS (see also Figure 5), 15.38% found their course structure similar, which was 12.47% less than the XPS group. However, around 50% claimed that adapting to the postgraduate course was easy, close to the figure (54.43%) in XPS. One possible reason is that when this online survey was conducted, the great majority of the master’s students were in the last several months of their postgraduate course, which illustrated that even they struggled during the adaptation process at the beginning. Consequently, they neither remained in the transitional period nor found it hard to adapt anymore. Moreover, Angela, a CPS participant from the London School of Economics (LSE), interpreted that “undergraduate and postgraduate curriculum was not the same system; initially we need to adjust, and then we can adapt them.” Notably, the number of postgraduates who chose the item “Different structure and content, but easy to adapt to” is the largest, and CPS is about 10% more than XPS. This gap reflects that the adaptability of CPS is better than XPS to some extent.

**Figure 6:**
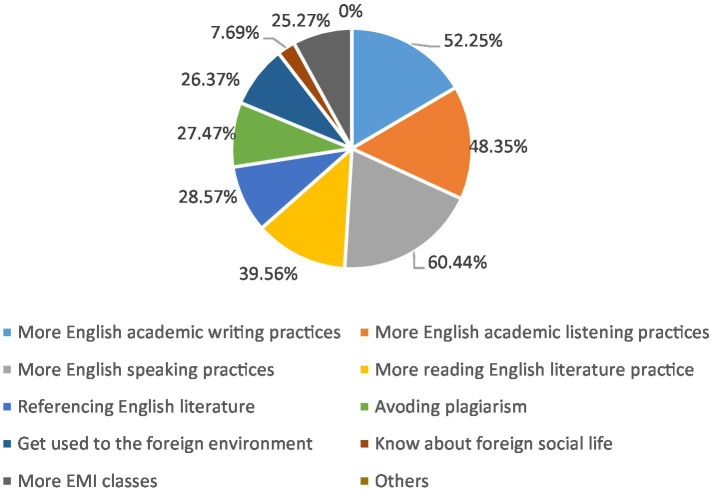
Things should have been improved in undergraduate study for CPS.

Language is another essential ability for students who expect to study in a foreign university, especially in classroom learning. Moreover, language barriers could negatively influence students’ input and output. In addition to English-related majors, most students mainly use Chinese during their undergraduate study in Double First-Class universities. They were asked to select the aspect that should be improved in their undergraduate study to help them better study in their postgraduate study. “More English speaking practices” (60.44%) and “More English academic writing practices” (52.25%) were the two most selected dimensions according to Figure 6. Jason from CPS stated that he did not learn academic writing during his undergraduate studies and could not utilize academic English to finish his postgraduate assignments. Furthermore, the exercises of undergraduate English are basically reading and CET-4 (China College English Test Band 4) or CET-6 (China College English Test Band 6); thus, speaking practice is even less. Admittedly, the student’s statements above may not apply to all the CPS, but to some extent, they signal that some CPS did not have enough chances to practice English speaking and know more about academic writing, demonstrating that CPS’s academic English skills are lacking in the undergraduate period.

**Figure 7:**
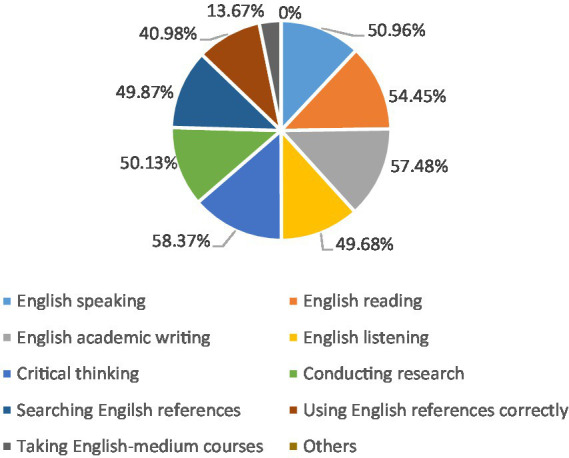
Skills gained from XJTLU.

As for XPS, they also frequently mentioned the significance and necessity of grasping English skills. One XPS in the Imperial College London said that if her cousins expect to study in the United Kingdom, she would highly suggest they learn English well now, especially improve communication in English. Figure 7 shows that English writing, reading, speaking, and listening abilities are all regarded as valuable skills acquired in XJTLU for postgraduates. Further, Lucy from XPS majoring in Management at the University of Oxford said: “All the classes from Year 2 to Year 4 were delivered in English, so after I came to Oxford, I think it is easy for me to adapt to, especially when I met British teachers, whose accent is quite familiar for me.” In addition to more than 50% of the students who selected four basic English skills (writing, speaking, listening, and reading), about half of the students chose critical thinking items, conducting research, and searching for English references. Moreover, XPS must take part in EAP academic English courses as a freshman, and this course covers academic English reading, academic writing, and speaking skills. Accordingly, their English academic skills can be improved through this course, and they can also apply their English skills to other subjects.

In conclusion, it is admitted that using English as a tool to study is vital by the two groups based on their own learning experiences. For CPS, many considered English language competence as one part that should have been improved in undergraduate study. While for XPS, the majority of them were already taking advantage of the language skills they obtained from XJTLU. After 4 years of learning in XJTLU, these students can keep up with the English teaching courses and know how to write academic English essays when studying in foreign countries. Consequently, the graduates of XJTLU may not have language barriers, which can guarantee better learning outcomes.

## Discussion

The above data indicate that two groups of students, XPS and CPS, showed different levels of learning satisfaction in postgraduate study. Specifically, it seems that a higher proportion of XPS reveals being satisfied with their learning outcomes than CPS, although these XPS generally scored less in College Entrance Examination than CPS. In addition, critical thinking and language skills play vital roles, as stated in the above section. The result shows that students who graduated from CPS for their undergraduate studies lacked critical thinking. This finding echoes Flowerdew’s (1998) finding that Chinese students lack critical thinking since they may experience more passive and rote learning before entering universities. Whereas the result of this study demonstrated that students from XPS have better critical thinking skills than students from CPS. This might be because XPS provides EMI context and EAP course, where critical thinking is seen as a defining characteristic (de Chazal, 2014; Zou and Jiang, 2021). Students can question and think critically in the EMI and EAP contexts. Consequently, if they were given a chance and encouraged to speak their mind, they could express various perspectives.

Furthermore, language competence is a necessity for postgraduate studies overseas, especially the academic English writing capability. As Counsell (2011) demonstrated, the learning outcomes are reflected in the forms of written assignments. Notably, the data in this study reveal that students from XPS (XJTLU) seemed to have fewer problems than CPS students in terms of English academic utilization. It is a high possibility that the EAP courses XPS took in Years One and Two developed their academic writing skills, as well as reading, writing, and listening skills, to be applied to other disciplines in the future. This indicates that students can improve their language skills in the EMI context before studying abroad for their postgraduate studies. EAP teaching and the EMI environment can help students have more competent in language skills. This finding is consistent with studies from Li and Ruan (2015) and Zou and Jiang (2021) that students at XJTLU developed well in English and academic skills. This result also corresponds to Soruç et al. (2022) and Zhang’s (2017) studies that academic English proficiency can help students have better learning performance in an EMI context. However, the result also shows that students from CPS had difficulties in language skills because they learned general English in their undergraduate studies, which is consistent with Hu’s (2014) study that Chinese students lacked language and academic writing skills, and general English skills are not very helpful to EFL learners learning outcomes in the EMI context (Curle et al., 2020). Thus, providing EMI instruction and EAP courses may help improve Chinese students’ critical thinking and language skills to cope with their postgraduate study abroad. This finding echoes Wu and Zhang’s (2017) study that EMI environment may help EFL students develop better writing skills than students in the non-EMI environment.

Moreover, two factors caused students’ differences in learning processes: gaps between tutors and curriculum design in undergraduate and postgraduate universities. Similarly, XPS is also faced with fewer gaps than CPS, as XJTLU adopts the British pedagogical system. Although there are some gaps, most CPS overcame the challenges successfully; thus, future research could be switched to CPS only and investigates how Chinese traditional Double First-Class university students overcome learning difficulties and achieved satisfactory results. In addition, participants’ responses, especially from those in the interviews, indicated that when investigating Chinese students’ learning satisfaction and outcomes, they tend to associate them more with the critical literacy skills, such as communication, critical thinking, and problem solving (Alfred et al., 1999), and less was mentioned to the other criteria, citizenship skills, including community involvement, intercultural understanding, and leadership. As a result, learning outcomes from Chinese postgraduates’ perspectives might be academic study centered.

In addition, these two groups have some similarities. Firstly, “knowledge gap between undergraduate and postgraduate study” is the common reason that affects their learning achievement. Students with professional knowledge in different fields would be more competitive than those with only one skill. In order to overcome this knowledge gap, self-learning and management abilities are essential, which are recognized by most interviewees. Students could relocate themselves to a new learning environment and manage their time between life and study.

## Conclusion

This study explored the impact of EMI education in China by comparing Chinese students’ learning outcomes in postgraduate studies in the United Kingdom and the United States. These students graduated from an EMI education: XJTLU (XPS), a Sino-foreign cooperative university and non-EMI education; Double First-Class universities (CPS) at the undergraduate level in China. The results show that language competence, critical thinking, self-learning, and self-management skills are three primary skills that participants agreed are vital in their postgraduate study. Overall, although CPS scored higher than XPS in College Entrance Examination, it seems that XPS might have a comparative higher learning satisfaction since features such as the educational system, curriculum design, and tutors’ teaching styles in XPS have more similarities to those in the United Kingdom and the United States. Since graduates in XJTLU acquire various skills from their undergraduate study, containing creative thinking, English academic skills, critical thinking, and environmental adaptability, XPS are able to quickly get their academic development in the United Kingdom and the United States, and they are more comfortable in the face of various challenges and difficulties in their postgraduate studies. Furthermore, XPS offered compulsory EAP courses in Years One and Two, laying an academic foundation for applying XPS in postgraduate study. Consequently, it is suggested that EAP courses could be introduced into Chinese universities for those who expect to study abroad and for students to publish papers in international journals.

Furthermore, through this comparative study of XJTLU and traditional Double First-Class university graduates in China, the competitiveness of XJTLU graduates has been reflected in diverse aspects. Although the scores of the undergraduates in the College Entrance Examination are not the highest, XJTLU’s educational philosophy holds that students may be able to develop their unlimited potential. Therefore, this research suggests that the innovation trend in higher education in China may contain more EMI context and EAP teaching to enhance internationalization and help Chinese students cope with their postgraduate studies in western countries. Moreover, this study enriches the literature on the theme of Sino-foreign cooperative universities and studying abroad in the context of China’s higher education reform. In conclusion, XJTLU’s achievements also provide a constructive reference for developing other Sino-foreign cooperative universities, creating a “student-centered” environment to help learners explore their interests and develop their potential independently.

Nevertheless, there are three limitations to this research. Firstly, the XPS group is a single Sino-foreign cooperative university. Thus, it may not represent Sino-foreign cooperative universities. More Sino-foreign cooperative universities should be included in future studies. Secondly, the choice of international students group comes from the United Kingdom and the United States; thus, it is possible to expand the range of countries to study, such as Australia and Canada, which will generate more precise data. Thirdly, other factors such as cross-cultural adaptation, parental support and university infrastructure should be also investigated in the future.

## Data availability statement

The datasets presented in this article are not readily available because: Data is used for the research only and cannot be distributed outside. Requests to access the datasets should be directed to bin.zou@xjtlu.edu.cn.

## Ethics statement

The studies involving human participants were reviewed and approved by Xi’an Jiaotong-Liverpool University. The patients/participants provided their written informed consent to participate in this study.

## Author contributions

BZ designed the study and conducted in-depth data analysis. XW conducted data analysis and drafted the whole paper. CY collected data and prepared the first draft. All authors contributed to the article and approved the submitted version.

## Conflict of interest

The authors declare that the research was conducted in the absence of any commercial or financial relationships that could be construed as a potential conflict of interest.

## Publisher’s note

All claims expressed in this article are solely those of the authors and do not necessarily represent those of their affiliated organizations, or those of the publisher, the editors and the reviewers. Any product that may be evaluated in this article, or claim that may be made by its manufacturer, is not guaranteed or endorsed by the publisher.
